# Genome-wide identification and characterization of active ingredients related β-Glucosidases in *Dendrobium catenatum*

**DOI:** 10.1186/s12864-022-08840-x

**Published:** 2022-08-23

**Authors:** Zhicai Wang, Meili Zhao, Xiaojie Zhang, Xuming Deng, Jian Li, Meina Wang

**Affiliations:** 1 Key Laboratory of National Forestry and Grassland Administration for Orchid Conservation and Utilization, Shenzhen, 518114 China; 2 Shenzhen Key Laboratory for Orchid Conservation and Utilization, The National Orchid Conservation Center of China and the Orchid Conservation & Research Center of Shenzhen, Shenzhen, 518114 China; 3 South China Limestone Plants Research Center, College of Forestry and Landscape Architecture, South China Agricultural University, Guangzhou, 510642 China; 4 Xinjiang Key Laboratory of Grassland Resources and Ecology, College of Grassland Sciences, Xinjiang Agricultural University, Urumqi, 830052 China

**Keywords:** *Dendrobium catenatum*, β-Glucosidase, Medicinal metabolites, Gene expression

## Abstract

**Background:**

*Dendrobium catenatum*/*D. officinale* (here after *D. catenatum*), a well-known economically important traditional medicinal herb, produces a variety of bioactive metabolites including polysaccharides, alkaloids, and flavonoids with excellent pharmacological and clinical values. Although many genes associated with the biosynthesis of medicinal components have been cloned and characterized, the biosynthetic pathway, especially the downstream and regulatory pathway of major medicinal components in the herb, is far from clear. β-glucosidases (BGLUs) comprise a diverse group of enzymes that widely exist in plants and play essential functions in cell wall modification, defense response, phytohormone signaling, secondary metabolism, herbivore resistance, and scent release by hydrolyzing β-D-glycosidic bond from a carbohydrate moiety. The recent release of the chromosome-level reference genome of *D. catenatum* enables the characterization of gene families. Although the genome-wide analysis of the *BGLU* gene family has been successfully conducted in various plants, no systematic analysis is available for the *D. catenatum*. We previously isolated *DcBGLU2* in the *BGLU* family as a key regulator for polysaccharide biosynthesis in *D. catenatum*. Yet, the exact number of *DcBGLU*s in the *D. catenatum* genome and their possible roles in bioactive compound production deserve more attention.

**Results:**

To investigate the role of *BGLU*s in active metabolites production, 22 *BGLU*s (*DcBGLU1*-*22*) of the glycoside hydrolase family 1 (GH1) were identified from *D. catenatum* genome. Protein prediction showed that most of the DcBGLUs were acidic and phylogenetic analysis classified the family into four distinct clusters. The sequence alignments revealed several conserved motifs among the DcBGLU proteins and analyses of the putative signal peptides and N-glycosylation site revealed that the majority of DcBGLU members dually targeted to the vacuole and/or chloroplast. Organ-specific expression profiles and specific responses to MeJA and *MF23* were also determined. Furthermore, four *DcBGLU*s were selected to test their involvement in metabolism regulation. Overexpression of *DcBGLU2*, *6*, *8*, and *13* significantly increased contents of flavonoid, reducing-polysaccharide, alkaloid and soluble-polysaccharide, respectively.

**Conclusion:**

The genome-wide systematic analysis identified candidate *DcBGLU* genes with possible roles in medicinal metabolites production and laid a theoretical foundation for further functional characterization and molecular breeding of *D. catenatum*.

**Supplementary Information:**

The online version contains supplementary material available at 10.1186/s12864-022-08840-x.

## Introduction

*Dendrobium*, one of the largest genera in the family Orchidaceae with approximately 1800 species worldwide [1], are popular economic plants owing to their beautiful flowers, scientific values and health benefits. Especially, *D. catenatum* is a highly prized *Dendrobium* species with a wealth of therapeutic functions in antitumor, anti-angiogenesis, anti-oxidation, anti-inflammation, diabetes alleviation, liver protection, stomach nourishing, body fluids supplementation, and immunity enhancement [2–5]. The stems of *D. catenatum* are the principal medicinal part containing large amounts of polysaccharides (> 30% of dry weight) and relatively low levels of ethanol extractives (~ 4.93% of dry weight), such as flavonoids, bibenzyls, phenanthrene and fluorenone [6]. The leaves, comprising approximately half of the total biomass of *D. catenatum*, are new sources of bioactive molecules, including phenolic compounds [7], flavonoids, polysaccharides, and amino acids [8]. The flowers of *D. catenatum* are rich with phenolic components (> 30% dry weight), while other substances such as essential and non-essential amino acids, polysaccharides, and volatile components are also found [9].

Many of the secondary metabolites are glucosylated to increase their solubility and stability, and activation of the glucosylated compounds is mediated by enzymes called β-glucosidases (BGLUs) [10]. BGLUs are groups of glycoside hydrolase 1 (GH1) family members found in all domains of living organisms with essential functions in removing nonreducing terminal glucosyl residues from glycosyl esters, oligosaccharides, and glycosides [10]. There are a great many glycosidases in higher plants with extensive redundant functions such as defense, cell wall remodeling, phytohormone activation, scent release, microbe/insect interactions, and secondary metabolism [11, 12]. In the last decades, genome-wide analysis of GH1-BGLUs has been carried out in a few plant species: *Arabidopsis thaliana* with 47 members (10 subfamilies), *Oryza sativa* with 40 members (8 subfamilies), *Zea mays* with 26 members (4 subfamilies), *Brassica rapa* with 64 members (10 subfamilies), and *Medicago truncatula* with 51 members (7 subfamilies) [13, 14]. For instance, a specific cytoplasmic BGLU has been demonstrated to hydrolyze the monoterpene alkaloid intermediate strictosidine to produce various monoterpene alkaloids [15]. Although some *BGLU*s related to metabolite biosynthesis were cloned and characterized from several plant species, limited information is available about *BGLU* family in orchids. Several *BGLU* genes expressed in the stems of three *Dendrobium* species (*Dendrobium huoshanese*, *D. catenatum*, and *Dendrobium moniliforme*) have been identified as hub genes that are possibly involved in polysaccharides biosynthesis [16]. In *Tongan vanilla*, β-glucosidase has been exogenously applied on green beans to hydrolyze glucovanillin into vanillin [17]. In *Cymbidium sinense*, *BGLU* genes are upregulated during ovule development, when β-glucosidase-mediated hydrolysis of cellulose may occur [18]. Fifteen *BGLU* genes are also significantly downregulated in *C. sinense* leaves, which may be an important reason for decreased starch content and abnormal sugar metabolism in the chloroplast [19]. *In D. catenatum*, *BGLU* genes are significantly upregulated in symbiotically germinated seeds [20], and *BGLU* genes associated with the glutathione metabolism pathway are upregulated in response to cadmium stress [21]. *In Dendrobium crumenatum*, a significant increase in β-glucosidase activity is observed during floral bud development [22].

Usually, the accumulation of medicinal metabolites, especially alkaloids, are low across tissues, making them hard to meet the threshold for drug-making [23]. Although genetic engineering and molecular modification are exceedingly helpful in creating improved varieties, the physiological and molecular mechanisms underlying metabolites production remain largely unexplored in *Dendrobium* plants [24]. Nevertheless, the great achievements in genome sequencing for *D. catenatum* [25, 26] make it convenient to conduct a genome-wide search for potential genes associated with important traits.

Previously, we sequenced the genome of *D. catenatum* [25] and reported *DcBGLU2* as a key regulator for polysaccharide accumulation in response to phytohormone treatments [27]. However, determining the exact number of *DcBGLU*s in *D. catenatum* genome and their corresponding roles in medicinal compounds production deserve more attention. The present study identified 22 candidate GH1 *DcBGLU*s members through a genome-wide analysis. Then their sequence feature, molecular phylogenetic relationship, conserved motif, gene structure, chromosomal localization, and *cis*-elements were characterized. Furthermore, the dynamic expression patterns in three more tissues were examined based on our and other published RNA-seq data. These results provide valuable information on the *DcBGLU* family in *D. catenatum* and lay a foundation for further exploring its function in plant metabolism.

## Materials and methods

### Genome-wide identification of GH1 BGLUs in *D. catenatum*

The genome sequences and protein data of the *D. catenatum* were downloaded from NCBI (https://www.ncbi.nlm.nih.gov/genome/?term=JSDN00000000) under the accession code JSDN00000000 [25]. To identify *D. catenatum* BGLU candidates of the GH1 family (DcBGLUs), the hidden Markov model (HMM) profile of Glyco_hydro_1 (PF00232) from the Pfam database (http://pfam.xfam.org/) were downloaded and searched against the BGLU domain in *D. catenatum* protein sequence data using the HMMER software (version 3.2.1, http://hmmer.org/download.html). After removing repeated or incomplete proteins, the remaining potential candidates were double-checked using the SMART (http://smart.embl-heidelberg.de/) and CDD (https://www.ncbi.nlm.nih.gov/Structure/bwrpsb/bwrpsb.cgi) databases. The gene identifiers used in this study are listed in the Table S1. The molecular weight (MW) and isoelectric point (pI) of each BGLU protein were calculated with the online ProtParam (https://web.expasy.org/protparam/) tool. The subcellular localization of each BGLU protein was predicted using the online ProtComp v. 9.0 server (http://www.softberry.com). The signal sequences were predicted using the SignalP (https://sFervices.healthtech.dtu.dk/service.php?SignalP-5.0) tool, and the N-glycosylation sites were detected by the NetNGlyc 1.0 server (https://services.healthtech.dtu.dk/service.php?NetNGlyc-1.0).

### Phylogenetic analysis of DcBGLUs

To classify and investigate phylogenetic relationships of BGLUs, the protein sequences from *A. thaliana* and *D. catenatum* were aligned using MEGA X [28]. The phylogenetic tree was constructed according to the neighbor-joining method with the bootstrap set at 1000 replicates, and then visualized and modified using EVOLVIEW (https://www.evolgenius.info/evolview/).

### Gene structures and conserved motifs

The exon/intron organization of *DcBGLU* genes was analyzed using the WebScipio server (https://www.webscipio.org/) [29]. The conserved motifs of DcBGLUs were identified by MEME suite (version 5.4.1) [30]. At the same time, the PlantCARE database (http://bioinformatics.psb.ugent.be/webtools/plantcare/html) was used to identify potential *cis*-elements in the 2000-bp gene promotors.

### RNA isolation and gene expression analysis

Approximately 300 mg of *D. catenatum* leaves transiently transformed with *BGLU*-*OE* plasmids, were homogenized in liquid nitrogen and then subjected to total RNA isolation using TRIzol reagent (Thermo Fisher Scientific) following the manufacturer’s instructions. RNA integrity was checked on a 1.5% agarose gel. Subsequently, 1.0 mg total RNA was reverse transcribed into first strand cDNA with a Prime Script™ reagent Kit with gDNA Eraser (TaKaRa, Japan), according to the manufacturer’s instructions. The cDNAs were subjected to a quantitative reverse transcriptase-polymerase chain reaction (qRT-PCR) using SYBR Green Premix Kit (Toyobo, Japan) in the ABI PRISM 7500 Fluorescent Quantitative PCR System (Thermo Fisher Scientific). All the primers used were listed in Table S2, with *Actin* as the internal control.

### Expression profiling of DcBGLUs from Transcriptomic data

Based on the transcriptome data from *D. catenatum* (GSE155403 and SRP150489) [31, 32], *D. huoshanense* (SRP122499) [33], and *Dendrobium nobile* (PRJNA338366) [34], the expressions of *DcBGLU*s and the corresponding orthologue genes were screened across four more tissues, mainly leaves, stems, roots, and protocorm-like body (PLBs), and under *MF23* treatment. All the raw SRA reads were transformed into fastq format, filtered, aligned, assembled, and estimated for expression levels. The results were visualized using heatmap generated from the TBtools [35].

### Cloning of DcBGLUs and transient transformation in *D. catenatum* leaves

The full-length coding sequences of *DcBGLU2*, *DcBGLU6*, *DcBGLU8*, and *DcBGLU13* were amplified using *D. catenatum* cDNA template with the primer sets listed in Table S3. The amplified fragments were cloned into pNC-Cam1304 binary vectors (NC biotech, Hainan, China) for gene overexpression. The corresponding constructs were then infiltrated into 2-year-old *D. catenatum* leaves following an *Agrobacterium*-mediated method [36] (Fig. S1).

### Measurement of the content of medicinal components

Contents of reducing-polysaccharides were determined following Tonukari et al.’s methods [37]. Contents of soluble-polysaccharides were measured using a plant soluble-polysaccharide assay kit (BC0035, Solarbio, Beijing, China) according to the manufacturer’s instructions. Contents of flavonoids were measured using a flavonoid assay kit (BC1335, Solarbio, Beijing, China) according to the manufacturer’s instructions. Contents of alkaloids were estimated following Wang et al.’s method [38]. Transiently transformed *D. catenatum* leaves (0.5 g) were harvested, grounded in liquid nitrogen, and used for extraction of compounds. The isolated compounds were determined by using a spectrophotometer (BeckmanCoulter DU730).

### Statistical analysis

For qRT-PCR gene expression analysis, *Actin* from *D. catenatum* was used as the internal control. The 2^-ΔΔCt^ method was used to calculate relative gene expression. Statistical significance is defined as follows: ** *p* < 0.01, *** *p* < 0.001, and **** *p* < 0.0001 (Student’s t-test).

## Results

### Genome-wide identification and characterization of BGLU genes in *D. catenatum*

The release and continuous update of the complete *D. catenatum* genome make it easier to conduct genome-wide identification of genes. A total of 22 candidate *DcBGLU* genes of the GH1 family were identified in the *D. catenatum* genome after strict HMMER screening and domain confirmation, and they were designated as *DcBGLU1* to *DcBGLU22* following the LOC accession numbers in the NCBI gene bank (Table S1). Among these 22 listed *DcBGLU*s, few were functionally characterized. For clarity, information on our previously published *DcBGLU*s was provided in Table S1 as well.

Physicochemical characteristics of the predicted DcBGLU proteins, including amino acid number, molecular weight, signal peptide, isoelectric point, GRAVY, N-gly site, and possible subcellular localization, are listed in Table 1. Approximately half of the predicted DcBGLU proteins (12/22) were predicted to have signal peptides ranging from 17 to 38 amino acids, targeting them to the secretory pathway. The length of the predicted precursor proteins varied between 245 aa (DcBGLU11) and 1050 aa (DcBGLU18), which correspond to protein molecular weight (MW) varied from 27.87 to 119.28 kDa. Most DcBGLU proteins contain one to ten N-linked glycosylation sites, except four DcBGLUs (DcBGLU11, 12, 16, and 21).

**Table 1:** Properties and locations of DcBGLUs in *D. catenatum*

Gene name	Accession	Amino acids	Molecular weight	Theoretical pI	GRAVY	Possible destination	Cleavage site	N-gly site
DcBGLU1	XP_020672326.1	514	58,375.15	7.08	− 0.285	Cyt,Chl,Vac	24-25	5
DcBGLU2	XP_020676273.1	529	60,188.07	5.31	−0.274	Cyt,Chl,Vac	25-26	1
DcBGLU3	XP_020676320.2	292	32,945.63	6.84	−0.327	Chl	–	4
DcBGLU4	XP_020676385.1	494	56,004.45	5.98	−0.264	Chl,Vac	17-18	2
DcBGLU5	XP_020676391.1	532	60,674.89	7.15	−0.357	Cyt,Chl,Vac	37-38	4
DcBGLU6	XP_020676421.1	519	59,111.99	5.84	−0.336	Cyt,Chl,Vac	22-23	4
DcBGLU7	XP_020680500.1	500	56,455.76	5.91	−0.207	Cyt,Chl,Vac	22-23	3
DcBGLU8	XP_020696227.1	509	57,758.21	5.79	−0.334	Chl,Vac	27-28	1
DcBGLU9	XP_020696243.1	517	58,633.17	5.57	−0.309	Chl,Vac	23-24	2
DcBGLU10	XP_020696596.1	524	59,773.52	6.47	−0.342	Cyt,Chl,Vac	25-26	4
DcBGLU11	XP_020697749.1	245	27,871.67	5.89	−0.136	Chl,Vac	–	–
DcBGLU12	XP_020699485.1	481	55,208.26	5.70	−0.387	Cyt,Chl,Vac	–	–
DcBGLU13	XP_020702441.1	305	33,725.20	8.30	0.022	–	–	3
DcBGLU14	XP_020704842.1	521	59,568.61	5.36	−0.209	Cyt,Chl,Vac	28-29	4
DcBGLU15	XP_020705021.1	504	56,828.32	6.12	−0.175	Cyt,Chl,Vac	22-23	3
DcBGLU16	XP_028548278.1	412	46,015.03	5.80	0.122	Chl	–	–
DcBGLU17	XP_028551772.1	411	47,565.02	7.68	−0.410	Chl,Vac	–	3
DcBGLU18	XP_028552937.1	1050	119,277.47	5.48	−0.222	Vac	25-26	10
DcBGLU19	XP_028555339.1	252	28,732.67	5.18	−0.145	–	–	3
DcBGLU20	XP_028556140.1	428	49,305.75	5.45	−0.576	Cyt,Chl,Vac	–	3
DcBGLU21	XP_028556317.1	436	50,660.65	7.07	−0.467	Chl,Vac	–	–
DcBGLU22	XP_028556369.1	424	48,466.92	5.72	−0.320	Chl,Vac	–	2

The theoretical isoelectric points (pI) of the predicted proteins varied widely from 5.18 (DcBGLU19) to 8.30 (DcBGLU13). DcBGLU5, 13, and 17 were basic proteins, DcBGLU1 and DcBGLU21 were neutral proteins, and the rest were acidic proteins (Table 1). The GRAVY ranged from − 0.576 to 0.022, suggesting that these DcBGLUs are all hydrophilic proteins. Additionally, most of the DcBGLUs were predicted to be in the vacuole, chloroplast and cytosol (10/22), chloroplast and/or vacuole (7/22). These results showed significant differences among the DcBGLU proteins, reflecting their diversified functions in *D. catenatum*.

### Phylogenetic analysis and classification of DcBGLU proteins

**Fig. 1:**
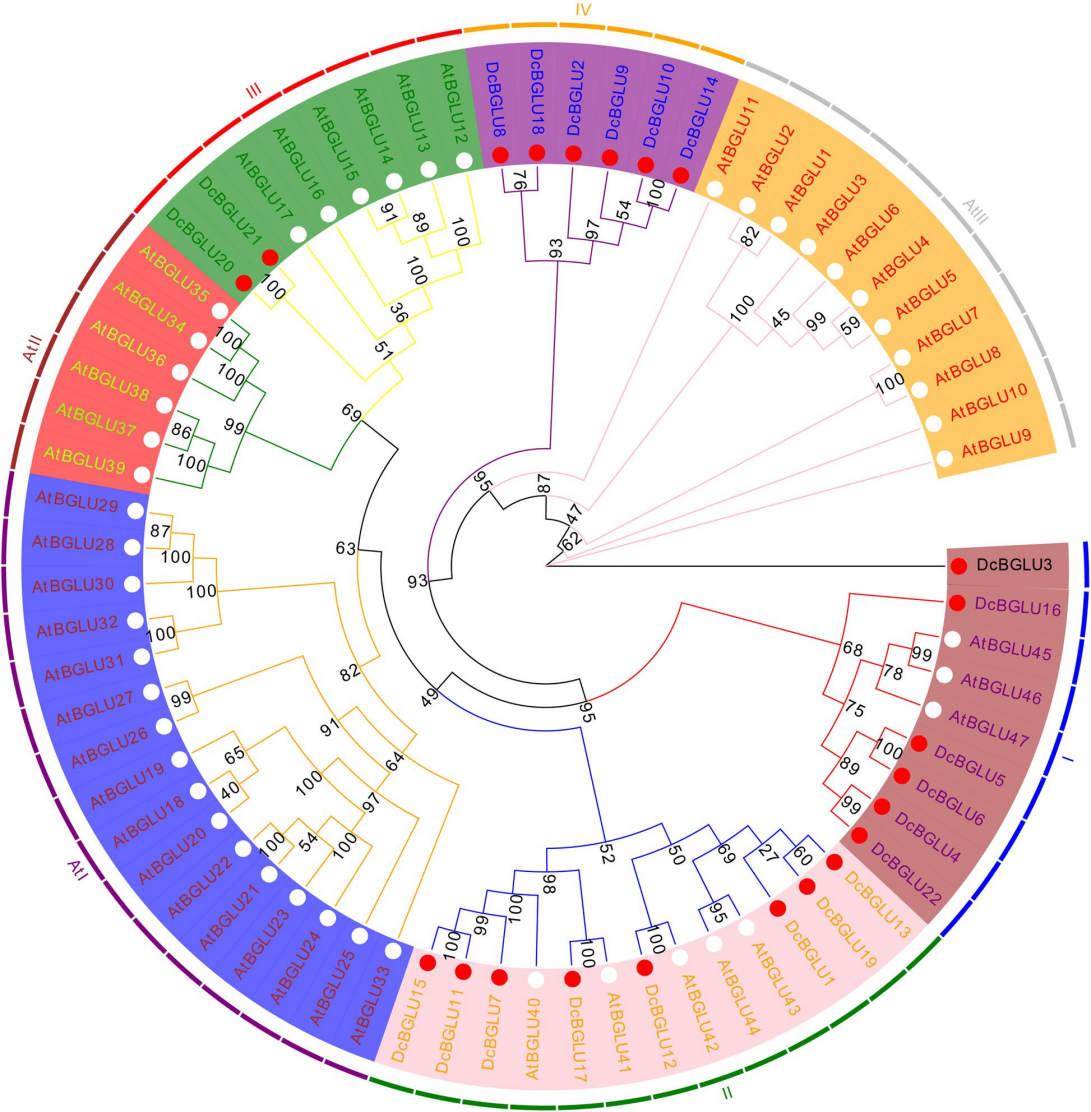
Phylogenetic relationship of the BGLUs from *D. catenatum* and *A. thaliana*. Phylogenetic tree of BGLUs using neighbor-joining (NJ) methods was constructed by MEGA X with 47 AtBGLU and 22 DcBGLU proteins. The subfamilies were marked in different colors. The identified DcBGLUs were highlighted by red circles

Multiple sequence alignment was conducted to further classify and characterize DcBGLU proteins, and results showed high conservation among the members (Fig. S2). Due to the high sequence similarity, the evolutionary relationship of DcBGLU proteins was investigated. Specifically, a sequence-based neighbor-joining phylogenetic tree was constructed for the proteins, including BGLU proteins both from *D. catenatum* and *A. thaliana*, using the MEGA X software (Fig. 1). The results from the phylogenetic tree showed that DcBGLUs could be divided into seven distinct clusters. Among the 22 DcBGLUs, 6, 8, and 2 belong to clusters I, II, and, III, respectively. While cluster IV contains six members only from *D. catenatum*, clusters AtI (16 members), AtII (6 members), and AtIII (11 members) contain members only from *A. thaliana*, suggesting that gene deletion could occur during the evolution of *D. catenatum*.

### Gene structure and conserved motif of DcBGLU proteins

**Fig. 2:**
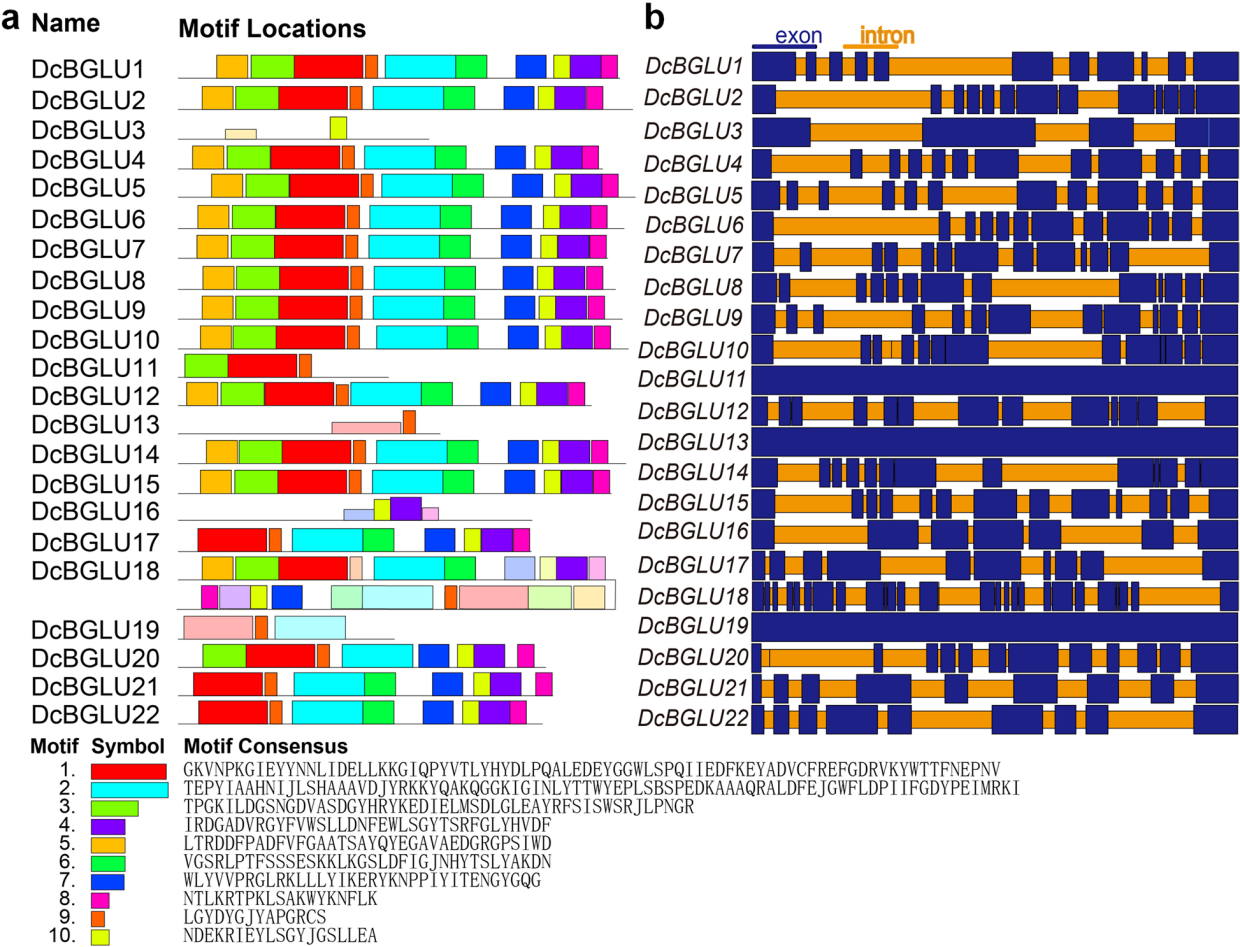
Architecture of conserved motifs and gene structures of DcBGLUs. **a** The motif composition and distribution of DcBGLU proteins. The colored boxes represent conserved motif and the grey lines indicate non-conserved lines. **b** Gene structures of *DcBGLU* genes. Exons and introns were indicated by blue rectangles and orange lines, respectively

To identify the conserved functional motifs in DcBGLU proteins, we searched using the MEME online tool and identified ten motifs. These conserved motifs possessed 14 to 82 amino acids, and the number of motifs varied from two to ten. The results showed that more than half of the members (13/22) possessed all these ten motifs and that motif two and nine were the two most conserved ones widely present in DcBGLU proteins (Fig. 2a). However, nine DcBGLU proteins lacked the complete combination of the conserved motifs, including five (DcBGLU3, 11, 13, 16 and 19) with less than four motifs, and the other four (DcBGLU17, 20, 21 and 22) have eight motifs. Nevertheless, members within the same cluster tend to share similar compositions of motifs, indicating the highly conservation between these DcBGLU proteins and the validity of cluster classification.

In order to better understand the evolutionary relationships of the GH1 family members in *D. catenatum*, exon/intron structures of all the identified *DcBGLU* genes were analyzed. The results showed that the exon numbers have varied considerably among the *DcBGLU* family members, from some having only one exon (*DcBGLU11*, *13*, and *19*) to some having up to 27 exons (*DcBGLU18*), including five with ten or less exons and thirteen with 11-13 exons (Fig. 2b). The exon/intron organization and intron numbers of the most closely related members in the same clusters were very similar.

### Promoter analysis and chromosomal distribution of *DcBGLU* genes

**Fig. 3:**
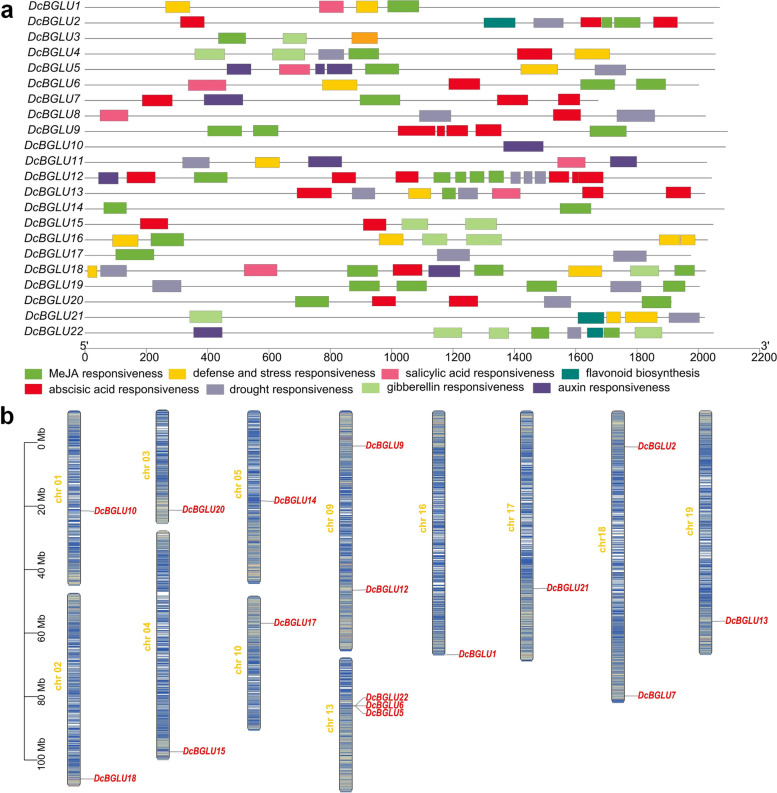
*Cis*-elements analysis and chromosomal localization of *DcBGLU*s. **a** *Cis*-elements in promoters of *DcBGLU* genes. Different colored wedges represented different *cis*-elements. **b** Chromosomal localization of *DcBGLU* genes

The members of different gene families could display diverse expression patterns due to functional divergence. The regulatory promoter often located upstream of the transcription initiation site of a gene has been recognized as one of the key factors in transcriptional regulation. In order to further investigate the potential regulatory mechanisms of *DcBGLU* genes in secondary metabolites production in response to environmental stimuli, about 2-kb upstream promoter regions of *DcBGLU* genes were submitted to the PlantCARE database for scanning the presence of key *cis*-acting elements. Three types of *cis*-regulatory elements, i.e., phytohormone-responsive, stress-responsive, and secondary metabolites biosynthesis (Flavonoid) elements, were detected (Fig. 3a). Firstly, five hormone-responsive elements, including MeJA, salicylic acid, abscisic acid, gibberellin, and auxin responses, were commonly presented in the promoter regions of the *DcBGLU* genes. Secondly, anaerobic, drought, low temperature, and defense and stress responsive elements were detected in these regions. Additionally, many light-responsive regulatory elements and MYB binding sites were widely present in these promoter regions (Supplementary File 1). Among these elements, MeJA responsive elements were the most common type of *cis*-regulatory elements (16/22) found in the promoters of *DcBGLU* genes (Fig. S3). This result supported the idea that accumulation of MeJA-induced secondary metabolites might be partially mediated by *DcBGLU*s. Moreover, flavonoid biosynthesis-associated *cis*-elements were detected in the promoters of *DcBGLU2*, *21*, and *22*, indicating the possible role of *DcBGLU*s in flavonoid production. The presence of *cis*-acting elements in the promoters of *DcBGLU* genes suggested that they might be responsible for adaptation to various stresses and hormone treatments by modulating the production of secondary metabolites.

The genomic distribution of *DcBGLU* genes was analyzed to provide an overview of the location on chromosomes. The 16 out of 22 *DcBGLU* genes were unevenly distributed on 12 of the 19 chromosomes (Fig. 3b). Most of the chromosomes have only one *DcBGLU* gene on each of them, except chromosome 9 with two members (*DcBGLU9* and *12*) and chromosome 13 with three members (*DcBGLU5*, *6*, and *22*). Interestingly, *DcBGLU5*, *6* and *22*, which belonging to the same subgroup I on the same chromosome 13, tended to cluster together, whereas the other family members were clustered separately.

### Organ and stress-specific expression patterns of the *DcBGLU* genes

**Fig. 4:**
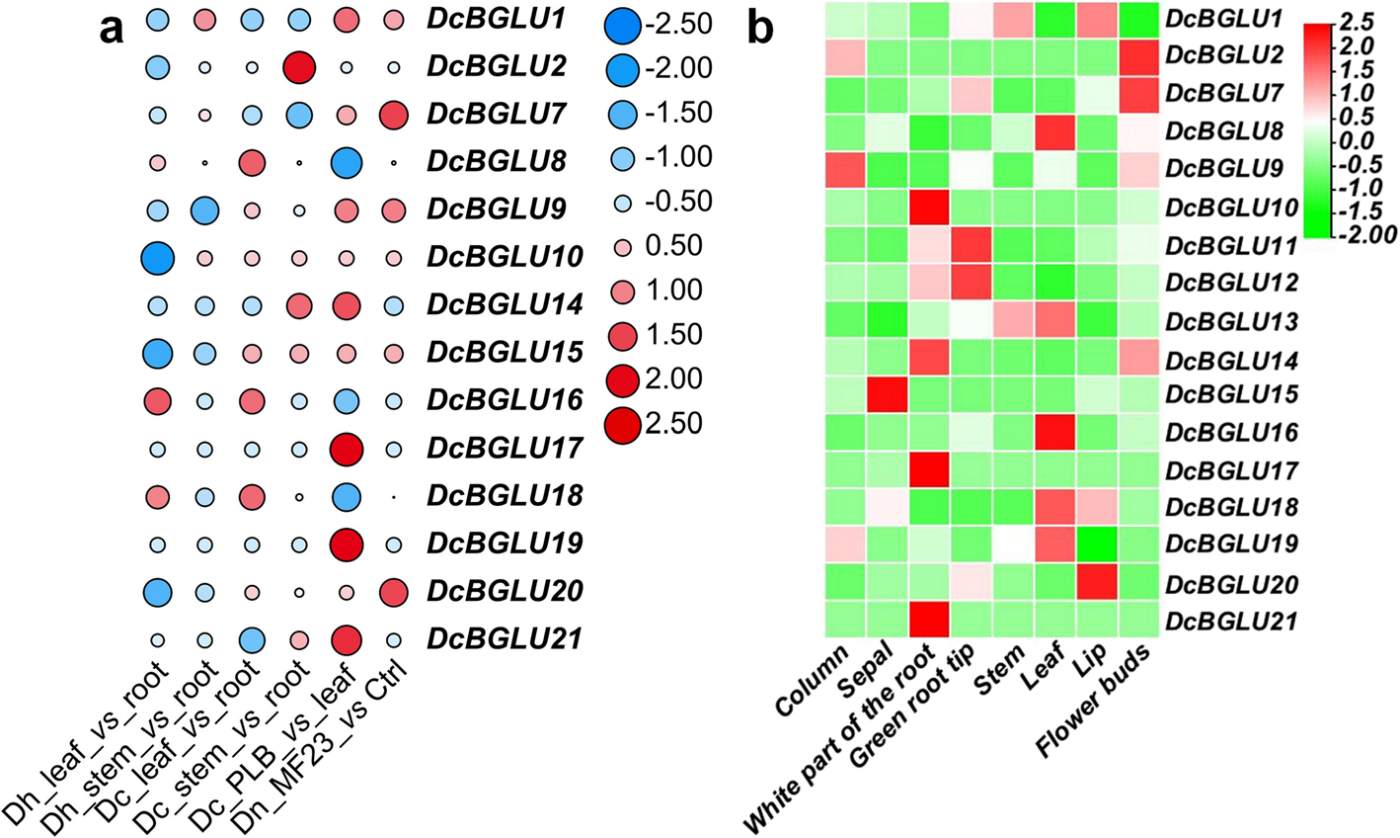
Expression profiles showing members of *DcBGLU*s in varied organs and different treatments. **a** Expression of *DcBGLU* genes in stem, leaf, PLB, and under *MF23* treatment. The log_2_(TPM values) of genes were shown by different color dots. Red and blue indicate high and low levels of expression, respectively. Each column indicates a treatment, and each row indicates a *DcBGLU* gene. **b** Expression patterns of *DcBGLU* genes in eight tissues. The expression levels of 17 *DcBGLU* genes were from the RNA-seq data. The eight samples included the column, sepal, stem, leaf, lip, flower bud, white root and green root tip. The color scale represents the values of log_2_(TPM value)

Plant *BGLU*s are well documented to play essential roles in response to developmental and environmental stimuli. Nevertheless, the functions of *BGLU*s in *D. catenatum* are far from clear. In the present study, four RNA-seq datasets of *D. catenatum* (GSE155403 and SRP150489) [31, 32], *D. huoshanense* (SRP122499) [33], and *D. nobile* (PRJNA338366) [34] were retrieved from NCBI Web Server, including samples across multiple tissues (root, stem, leaf, and PLB) and samples infected with *MF23* (Fig. 4a). Overall, 14 differentially expressed *DcBGLU*s were identified in *D. catenatum*, among which *DcBGLU8*, *16*, and *18* were more highly expressed in leaves than in roots. However, *DcBGLU2* and *14* were more highly expressed in stems than in roots. The expression analysis indicated that four *BGLU* orthologue genes corresponding to *DcBGLU1*, *7*, *9*, and *20*, respectively, in *D. nobile* were induced by *MF23* infection and that the expression patterns of corresponding genes expressed in leaves or stems vs roots in *D. catenatum* and *D. huoshanense* are very similar.

Because of the close relevance between gene expression and function, the expression profiles of *DcBGLU*s in eight tissues (column, sepal, white root, green root tip, stem, leaf, lip, and flower bud) from RNA-seq datasets (PRJNA348403) [39] were analyzed. *DcBGLU*s showed different transcriptional patterns across various tissues (Fig. 4b). Some *DcBGLU*s are specifically expressed in floral organs (e.g., *DcBGLU9* in the column, *DcBGLU1* and *20* in the lip, *DcBGLU2*, *7*, and *14* in the flower buds), suggesting their potential role in pigmentation and scent release. For *DcBGLU10*, *11*, *12*, *14*, *17*, and *21*, they tended to be more highly expressed in white roots and green root tips than in other tissues. Additionally, the *DcBGLU*s classified in the same cluster did not always have the same expression pattern. For example, compared with the members of *DcBGLU2*, *9*, *10* and *14* in cluster IV, *DcBGLU8* and *18* from the same clade were more highly expressed in leaves. These results indicated that the expression pattern of *DcBGLU* genes was diverse and tissue-specific.

### Functional validation of DcBGLU genes in medicinal compounds accumulation

**Fig. 5:**
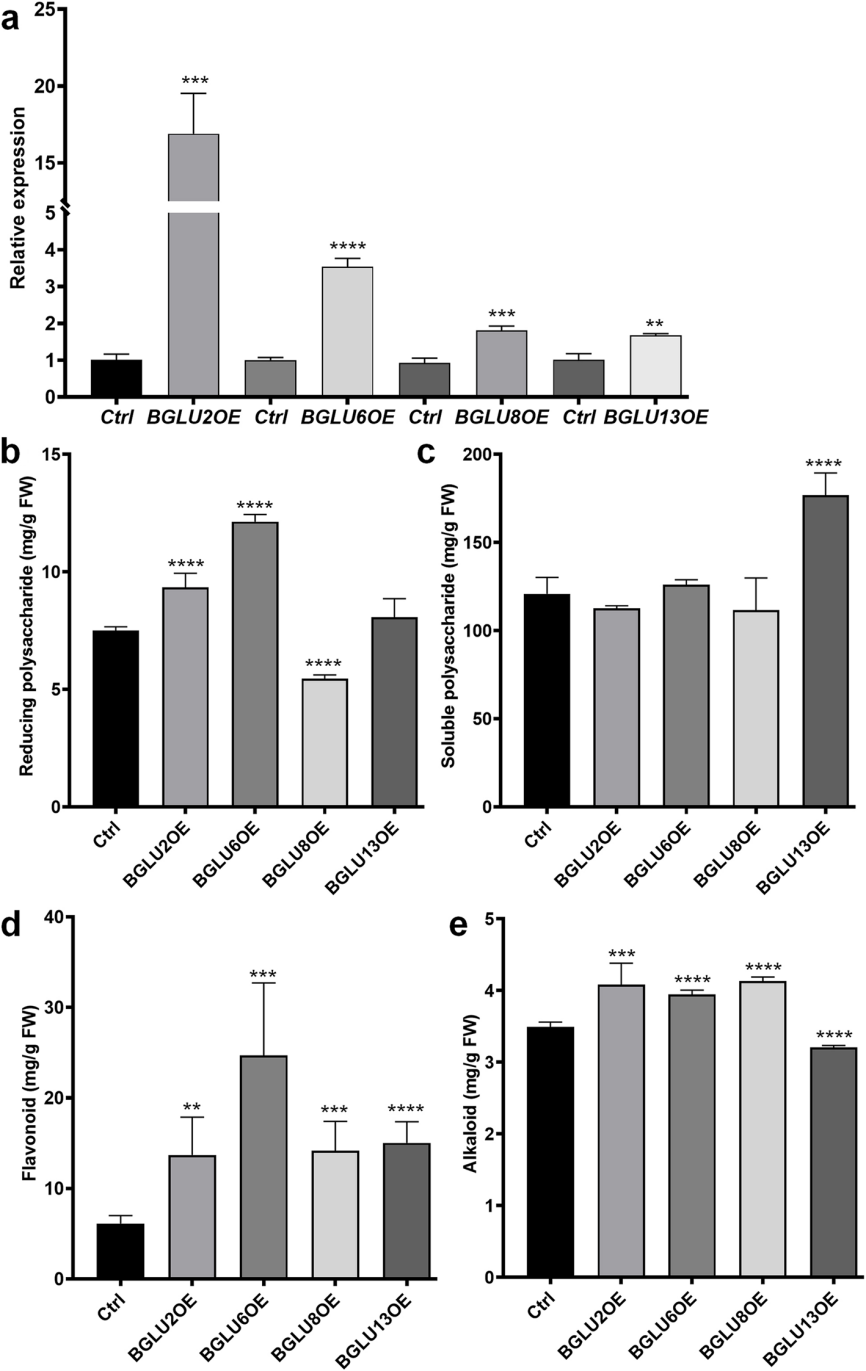
Overexpression of *DcBGLU*s altered major medicinal metabolites accumulation. **a** qRT-PCR verification of the overexpression status of four selected *DcBGLU* genes in transiently transformed *D. catenatum* leaves. Samples transformed with empty vectors were used as the controls (Ctrl). **b-e** Determination of major medicinal metabolite contents in *BGLU*-*OE* leaves. **b** Reducing-polysaccharide content; **c** Soluble-polysaccharide content; **d** Flavonoid content; **e** Alkaloid content. Leaves transiently transformed with *Agrobacterium EHA105* carrying empty vector were used as control. Bars marked with stars indicate significant differences (Student’s t-test, * *p* < 0.05, ** *p* < 0.01, *** *p* < 0.001, **** *p* < 0.0001)

As our previous study suggested that the expression of *DcBGLU2* was closely related to reducing-polysaccharide production [27], we thus verified the function by transient overexpression of this gene in *D. catenatum* leaves. Upregulated expression was detected in the infiltrated leaves 6 hours later (Fig. 5a), with accumulated levels of reducing-polysaccharides, flavonoids, and alkaloids 5 days later. We also overexpressed *DcBGLU8*, another member in the same cluster as *DcBGLU2*. Results showed that overexpression of *DcBGLU8* had similar promoting roles as *DcBGLU2* in flavonoids and alkaloids accumulation but somehow suppressed reducing-polysaccharide production (Fig. 5b**)**. Besides, we also tested the possible functions of the members from the other two clusters, *DcBGLU6* from cluster I and *DcBGLU13* from cluster II, in medicinal metabolites production. Overexpression of *DcBGLU6* greatly induced accumulation of reducing-polysaccharides and flavonoids, and slightly but significantly increased accumulation of alkaloids. Meanwhile, overexpression of *DcBGLU13* enhanced the production of soluble-polysaccharides (Fig. 5c**)** and flavonoids, but slightly reduced alkaloids (Fig. 5d, e**)**. These results reflected the diversified functions of *DcBGLU*s in regulating medicinal metabolites accumulation in *D. catenatum*.

## Discussion

*Dendrobium* orchids are highly prized medicinal herbs that have been widely used for many years. The plant contains various bioactive components, including polysaccharides, flavonoids, and alkaloids [40]. Several transcriptome profiles have been conducted in *Dendrobium* species to reveal the putative genes and pathways involved in active metabolites biosynthesis [25, 41, 42]. Moreover, genome-wide characterization of gene families has also begun to shed light on the biosynthesis pathways in several plant species [43]. Glucosidases are multifunctional enzymes that play crucial roles in plant development, secondary metabolites biosynthesis, and responses to biotic and abiotic stresses [11, 12]. Recently, the genome-wide characterization of *BGLU* members in different plant species has been performed with the rapid advancements of whole-genome sequencing technologies. Compared with the other plant species, the number of CH1-*BGLU* genes in *Orchidaceae* (*C. sinense*, *Apostasia shenzhenica*, *Vanilla shenzhenica*, *Phalaenopsis equestris*, *Phalaenopsis aphrodite* and *D. catenatum*) is much smaller, suggesting that there may be gene loss or pseudogenization in the process of evolution [19]. Nevertheless, in *D. catenatum*, a systematic analysis of *BGLU* family members has not been available despite being one of the most important medicinal orchids and the importance of *BGLU* genes in plant secondary metabolism. The recent availability of high-quality annotated reference genome [25] provides valuable resources for studying the BGLU family in *D. catenatum*. This study, to our knowledge, provides the first report concerning the systematic analysis and functional role of *BGLU* family genes from *D. catenatum* on secondary metabolism. This study identified 22 full-length *BGLU* genes from the *D. catenatum* genome, 16 of which were unevenly distributed on 12 chromosomes. We also analyzed the phylogenetic relations of *DcBGLU*s and evaluated gene expression patterns in different tissues and under biotic stress. In addition, correlation analysis between gene expression and metabolites content verified four candidate genes involved in active metabolites biosynthesis. Overall, the current study comprehensively investigated and presented the *BGLU* genes in *D. catenatum*. This endeavor will be beneficial for the in-depth exploration of biological functions of the *BGLU* gene family and provide potential targets for molecular breeding.

The current study identified 22 *DcBGLU* genes throughout the *D. catenatum* genome and characterized them using multi-sequence alignment and phylogenetic analysis. The results showed that the *DcBGLU*s shared high sequence similarity and conserved domain during evolution. *DcBGLU*s were mainly targeted into cytosol, chloroplast, and vacuole, where they can access the physiological substrates for catalysis. Besides, most *DcBGLU*s contain at least one predicted N-glycosylation site, corresponding to the estimation that many of *DcBGLU*s might hydrolyze their substrates through secretory pathways.

Jasmonate (JA) is a plant-specific signaling molecule broadly associated with the biosynthesis of various secondary metabolites. The exogenous application of methyl jasmonate (MeJA) has been frequently used to manipulate the production of polysaccharides [44] and alkaloids in *D. catenatum* [45]. In the present study (Fig. 3a), we prove that MeJA-responsive *cis*-elements are widely distributed in most of *DcBGLU* promoters (16/22), suggesting that *DcBGLU*s might partially mediate MeJA-induced metabolites accumulation. Salicylic acid (SA) is another stress signaling molecule that can be used as an elicitor to promote the biosynthesis of plant secondary metabolites [46]. Both MeJA and SA have commonly been used in various plant cultures, including PLB, callus, shoot, and root culture systems [47, 48]. The promoters of *DcBGLU* members, including *DcBGLU1*, *5*, *6*, *11*, *13*, and *18*, have SA-responsive elements, implying their possible roles in elicitor-induced active metabolites accumulation.

Even though plant-derived alkaloids are beneficial to human health, for plants, alkaloids are essential for defensive responses to environmental stresses. For example, binary stress can increase indole alkaloid levels in *Catharanthus roseus* [49], drought stresses can increase the accumulation of alkaloid in roots of motherwort (*Leonurus japonicas*) [50], *Ceratocystis fimbriata* infection can enhance alkaloids production in mango (cultivar Ubá) [51], and *MF23* infection can increase alkaloids accumulation in *D. nobile* [34]. Accordingly, many defense and stress responsive elements presented in the promoter regions of *DcBGLU*s. *MF23* induced *BGLU1*, *7*, *19*, and *20* expressions (Fig. 4a) with increased dendrobine alkaloid levels [34]. Anthocyanin flavonoids, normally present as glycosides, are the main determinants of flowers colors [52]. Tea made from *D. catenatum* flowers has been consumed for many years, in which flavonoids might be one of the major beneficial ingredients. *DcBGLU1*, *2*, *7*, *9*, *14*, and *20* were highly expressed in *D. catenatum* floral organs (Fig. 4b), indicating their possible functions in flavonoid biosynthesis.

Stem-specific expressions of *AtBGLU45* and *AtBGLU46* in *A. thaliana* are responsible for hydrolysis of lignin precursors in response to various stresses [53]. Grouped in the same cluster (i.e., cluster I), however, *DcBGLU16* was mainly expressed in leaves of *D. catenatum* (Fig. 4b). Root-specific *AtBGLU42* has been known to modulate rhizobacteria-induced systematic resistance in *A. thaliana* [54]. Even though *DcBGLU12* in the same clade (i.e., clade II) expressed in roots, it failed to respond to *MF23* infection as revealed by the RNA-seq analysis (Fig. 4a), indicating the diversified functions between *AtBGLU*s and *DcBGLU*s.

Plant BGLUs are groups of GH1 enzymes that remove the nonreducing terminal β-D-glucosyl residue from glucoconjugates. BGLU enzymes contribute to various biological functions in plants, including cell wall remodeling, defense and stress response, scent release, phytohormone activation, and secondary metabolism [55]. BGLUs can hydrolyze metabolic intermediates to release glucosyl blocking groups and allow further metabolization to various natural products, many of which are medicinally relevant compounds. For example, the monoterpene alkaloid strictosidine is hydrolyzed by a specific cytoplasmic β-glucosidase to produce various monoterpene alkaloids [15, 56]. In addition to hydrolysis activity, many BGLUs also showed transglucosidase activities for synthesizing glucoconjugates, such as anthocyanin biosynthesis in *Agapanthus africanus* and *A. thaliana* [57, 58]. This glycosylation and deglycosylation process perfectly maintained the homeostasis of plant metabolites. In a previous study, we found a close association between the expression of *DcBGLU2* and polysaccharide content in *D. catenatum* [27], suggesting that the *DcBGLU2* might work as a transglucosidase in polysaccharide biosynthesis. Consistent with these findings, overexpression of *DcBGLU2* increased the reducing-polysaccharides, flavonoids, and alkaloids in *D. catenatum* (Fig. 5). Moreover, *SENSITIVE TO FREEZING 2* (*SFR2*) encoded enzymes from carnation (*Dianthus caryophyllus*), delphinium (*Delphinium grandiflorum*), and *A. taliana* can transglycosylate monogalactosyl diacylglyceride to di-, tri-, and tetra-galactosyl diacylglycerides [59, 60]. The present study also identified *DcBGLU3* and *DcBGLU16* as *BGLU-like SFR2* genes in cluster I (Table S1), indicating similar functions between them. Overexpression of exogenous β-glucosidase in tobacco chloroplasts increased phytohormone levels and thus the resistance to white flies and aphids [61]. Likewise, the transformation of the same *BGLU* into *Artemisia annua* vacuole increased trichome numbers and artemisinin production [62]. These works showed the potential application of manipulating β-glucosidase in plants to prompt medicinal metabolites production.

## Conclusion

The present study has investigated the classification and expression profile of 22 GH1 *DcBGLU*s in different tissues and under stress conditions. Although some family members of *AtBGLU*s and *DcBGLU*s shared high sequence similarities, the tissue and stress responsiveness were diversified. Therefore, the present study paves the way for further dissection of the distinct role of *DcBGLU*s in secondary metabolism and other functions.

## Supplementary Information

**Additional file 1.**

**Additional file 2: Fig. S1.** Transient expression of *DcBGLU*s in leaves of *D. catenatum.* **Fig. S2.** Amino acid sequence alignment of 22 DcBGLU enzymes in *D. catenatum.* **Fig. S3.** Analysis of the numbers and types of *cis*-acting elements in *DcBGLU* genes. **Table S1.** Accession numbers for proteins used in the phylogenetic tree. **Table S2.** Primers used for qRT-PCR validation. **Table S3.** Primers used for gene cloning. **Table S4.** Abbreviations.

## Data Availability

The genome sequences and protein data of the *D. catenatum* were downloaded from NCBI (https://www.ncbi.nlm.nih.gov/genome/?term=JSDN00000000) under the accession code JSDN00000000 [25]. Transcriptome data from *D. catenatum* (GSE155403 and SRP150489) [31, 32], *D. huoshanense* (SRP122499) [33], and *D. nobile* (PRJNA338366) [34] were used for *BGLU* expression profiling.
