# Supplementary material for: Genome-wide identification and characterization of active ingredients related β-Glucosidases in Dendrobium catenatum
Source: BMC Genomics. 2022 Aug 23;23:612. doi: 10.1186/s12864-022-08840-x (PMC9400273; doi:10.1186/s12864-022-08840-x)
Supplement: Supplementary file 2 — Additional file 2: Fig. S1. Transient expression of DcBGLUs in leaves of D. catenatum. Fig. S2. Amino acid sequence alignment of 22 DcBGLU enzymes in D. catenatum. Fig. S3. Analysis of the numbers and types of cis-acting elements in DcBGLU genes. Table S1. Accession numbers for proteins used in the phylogenetic tree. Table S2. Primers used for qRT-PCR validation. Table S3. Primers used for gene cloning. Table S4. Abbreviations. [file 12864_2022_8840_MOESM2_ESM.zip › Supporting Information.pdf]

Supporting Information

Genome-wide identification and characterization of active ingredients related β-Glucosidases in *Dendrobium catenatum*

Zhicai Wang [1,2]*, Meili Zhao[1,2,3], Xiaojie Zhang[1,2,4], Xuming Deng, Jian Li[1,2], Meina Wang[1,2]*

[1] Key Laboratory of National Forestry and Grassland Administration for Orchid Conservation and Utilization, Shenzhen 518114, China; 1522830227@qq.com (M.Z.); 2428815613@qq.com (X.Z.); 798731337@qq.com (X.D.); etecology@foxmail.com (J.L.)

[2] Shenzhen Key Laboratory for Orchid Conservation and Utilization, The National Orchid Conservation Center of China and the Orchid Conservation & Research Center of Shenzhen, Shenzhen 518114, China

[3] South China Limestone Plants Research Center, College of Forestry and Landscape Architecture, South China Agricultural University, Guangzhou 510642, China

[4] Xinjiang Key Laboratory of Grassland Resources and Ecology, College of Grassland Sciences, Xinjiang Agricultural University, Urumqi 830052, China

*Correspondence: wzcxjnu.5525@163.com (Z.W.); 328365210@qq.com (M.W.)

Table S1. Accession numbers for proteins used in the phylogenetic tree

Protein name	Protein ID	Functional annotation	Previously named [1]
DcBGLU1	XP_020672326.1	beta-glucosidase 1-like	DcBGLU1
DcBGLU2	XP_020676273.1	beta-glucosidase 2-like	DcBGLU2L
DcBGLU3	XP_020676320.2	beta-glucosidase-like SFR2	--
DcBGLU4	XP_020676385.1	beta-glucosidase 18 isoform X2	--
DcBGLU5	XP_020676391.1	beta-glucosidase 18 isoform X1	DcBGLU18
DcBGLU6	XP_020676421.1	beta-glucosidase 18 isoform X1	--
DcBGLU7	XP_020680500.1	beta-glucosidase 6-like	DcBGLU6
DcBGLU8	XP_020696227.1	beta-glucosidase 22	--
DcBGLU9	XP_020696243.1	beta-glucosidase 22	--
DcBGLU10	XP_020696596.1	beta-glucosidase 2	DcBGLU2
DcBGLU11	XP_020697749.1	beta-glucosidase 34-like	DcBGLU34
DcBGLU12	XP_020699485.1	beta-glucosidase 4	DcBGLU4
DcBGLU13	XP_020702441.1	receptor-like protein kinase	--
DcBGLU14	XP_020704842.1	beta-glucosidase 22	--
DcBGLU15	XP_020705021.1	beta-glucosidase 6	--
DcBGLU16	XP_028548278.1	beta-glucosidase-like SFR2	SFR2
DcBGLU17	XP_028551772.1	putative beta-glucosidase 41	DcBGLU41
DcBGLU18	XP_028552937.1	lactase-phlorizin hydrolase	DcBGLU22
DcBGLU19	XP_028555339.1	beta-glucosidase 1-like, partial	--
DcBGLU20	XP_028556140.1	beta-glucosidase 11-like	DcBGLU11L
DcBGLU21	XP_028556317.1	furostanol glycoside 26-O-beta-glucosidase-like	DcBGLU12
DcBGLU22	XP_028556369.1	beta-glucosidase 18	--
AtBGLU1	NP_849771.2	*Arabidopsis thaliana* beta glucosidase 1	
AtBGLU2	NP_197161.2	*Arabidopsis thaliana* beta glucosidase 2	
AtBGLU3	NP_193941.2	*Arabidopsis thaliana* beta glucosidase 3	
AtBGLU4	NP_176217.2	*Arabidopsis thaliana beta glucosidase 4*	
AtBGLU5	NP_001319275.1	*Arabidopsis thaliana* beta glucosidase 5	
AtBGLU6	NP_176233.2	*Arabidopsis thaliana* beta glucosidase 6	
AtBGLU7	NP_191833.2	*Arabidopsis thaliana* beta glucosidase 7	
AtBGLU8	NP_001327941.1	*Arabidopsis thaliana* beta glucosidase 8	
AtBGLU9	NP_194511.3	*Arabidopsis thaliana* beta glucosidase 9	
AtBGLU10	NP_567787.1	*Arabidopsis thaliana* beta glucosidase 10	
AtBGLU11	NP_001117217.1	*Arabidopsis thaliana* beta glucosidase 11	
AtBGLU12	NP_199041.1	*Arabidopsis thaliana* beta glucosidase 12	
AtBGLU13	NP_199277.1	*Arabidopsis thaliana* beta glucosidase 13	
AtBGLU14	NP_850065.1	*Arabidopsis thaliana* beta glucosidase 14	
AtBGLU15	NP_181973.1	*Arabidopsis thaliana* beta glucosidase 15	
AtBGLU16	NP_191572.1	*Arabidopsis thaliana* beta glucosidase 16	
AtBGLU17	NP_001324585.1	*Arabidopsis thaliana* beta glucosidase 17	

AtBGLU18	NP_175649.1	*Arabidopsis thaliana* beta glucosidase 18
AtBGLU19	NP_188774.2	*Arabidopsis thaliana* beta glucosidase 19
AtBGLU20	NP_177722.1	*Arabidopsis thaliana* beta glucosidase 20
AtBGLU21	NP_176801.1	*Arabidopsis thaliana* beta glucosidase 21
AtBGLU22	NP_176802.1	*Arabidopsis thaliana* beta glucosidase 22
AtBGLU23	NP_187537.1	*Arabidopsis thaliana* beta glucosidase 23
AtBGLU24	NP_198203.1	*Arabidopsis thaliana* beta glucosidase 24
AtBGLU25	NP_187014.1	*Arabidopsis thaliana* beta glucosidase 25
AtBGLU26	NP_181977.1	*Arabidopsis thaliana* beta glucosidase 26
AtBGLU27	NP_191571.4	*Arabidopsis thaliana* beta glucosidase 27
AtBGLU28	NP_850416.1	*Arabidopsis thaliana* beta glucosidase 28
AtBGLU29	NP_001118524.1	*Arabidopsis thaliana* beta glucosidase 29
AtBGLU30	NP_191573.1	*Arabidopsis thaliana* beta glucosidase 30
AtBGLU31	NP_197842.1	*Arabidopsis thaliana* beta glucosidase 31
AtBGLU32	NP_197843.2	*Arabidopsis thaliana* beta glucosidase 32
AtBGLU33	NP_180845.2	*Arabidopsis thaliana* beta glucosidase 33
AtBGLU34	NP_175191.2	*Arabidopsis thaliana* beta glucosidase 34
AtBGLU35	NP_175558.3	*Arabidopsis thaliana* beta glucosidase 35
AtBGLU36	NP_001319196.1	*Arabidopsis thaliana* beta glucosidase 36
AtBGLU37	NP_568479.1	*Arabidopsis thaliana* beta glucosidase 37
AtBGLU38	NP_851077.1	*Arabidopsis thaliana* beta glucosidase 38
AtBGLU39	NP_680406.1	*Arabidopsis thaliana* beta glucosidase 39
AtBGLU40	NP_173978.1	*Arabidopsis thaliana* beta glucosidase 40
AtBGLU41	NP_200268.3	*Arabidopsis thaliana* beta glucosidase 41
AtBGLU42	NP_001031975.1	*Arabidopsis thaliana* beta glucosidase 42
AtBGLU43	NP_188435.2	*Arabidopsis thaliana* beta glucosidase 43
AtBGLU44	NP_188436.1	*Arabidopsis thaliana* beta glucosidase 44
AtBGLU45	NP_176374.1	*Arabidopsis thaliana* beta glucosidase 45
AtBGLU46	NP_850968.1	*Arabidopsis thaliana* beta glucosidase 46
AtBGLU47	NP_001328261.1	*Arabidopsis thaliana* beta glucosidase 47

Table S2. Primers used for qRT-PCR validation

Genes	Name	Sequences
BGLU2	Forward	5' CTTTCAGCAAATCAGAATCCCA 3'
	Reverse	5' ATGCCTGACTGGTTGGTGGA 3'
BGLU6	Forward	5' GTACTTTAGAAGGCTATGGATTTGC 3'
	Reverse	5' AAGCCACTTGGAACCACATAGA 3'
BGLU8	Forward	5' TCTAAATGAAACAAGTGAGGGTCC 3'
	Reverse	5' TGGCTCCCAACCCATAACC 3'
BGLU13	Forward	5' ACATAACGCATGGAGACAGAGTG 3'
	Reverse	5' GTGGGCAATAATGTAAGGTTCTG 3'
ACTIN	Forward	5' GAAGCCCAGTCCAAAAGAGGTATCC 3'
	Reverse	5' ACATGGCAGGCACATTGAAAGTCTC 3'

Table S3. Primers used for gene cloning

Genes	Name	Sequences
BGLU2	Forward	5' ATGGGGACTCCCATTGTCTTAC 3'
	Reverse	5' TCATCCACTGAAAAAGCCACC 3'
BGLU6	Forward	5' ATGAAGGGAAATGAAGAAGCTATTG 3'
	Reverse	5' TTACTGTGCCACCAGCTTTGG 3'
BGLU8	Forward	5' ATGGCTGCCTCATCTCTACGC 3'
	Reverse	5' TCACTGTGAAGCAAGGGATTTT 3'
BGLU13	Forward	5' ATGCCTGACCTCACACTTATGTG 3'
	Reverse	5' CTATACTTTCTCTGCAGTAGTATGAAGTAAGA 3'

Table S4. Abbreviations

**Full name**	**Abbreviations**
β-glucosidases	BGLU
Dendrobium catenatum	*D. Catenatum*
[redacted]	qRT-PCR
Jasmonate	JA
Methyl Jasmonate	MeJA
Protocorm-like bodies	PLB
glycoside hydrolase family 1	GH1
Salicylic acid	SA
Molecular weight	MW
Amino acid	aa
Isoelectric point	pI

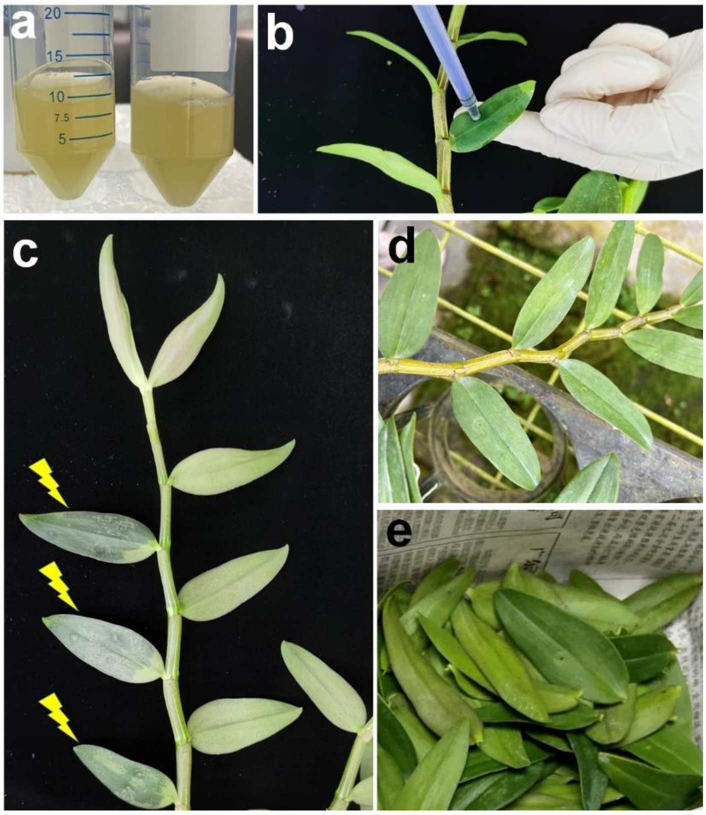

Figure S1. Transient expression of *DcBGLU*s in leaves of *D. catenatum*. **(a)** Prepared *Agrobacterium* carrying empty vectors (*EV*) or *DcBGLU* overexpression (*OE*) plasmids for leaf infiltration. **(b)** Transient leaf infiltration with syringe (1 mL in size). **(c)** A closer look of the infiltrated leaves (Marked with lightning) in comparison with the non-infiltrated ones. **(d)** Infiltrated leaves were led to grow at greenhouse for another 5 days. **(e)** Infiltrated leaves were collected for medicinal compound measurement.

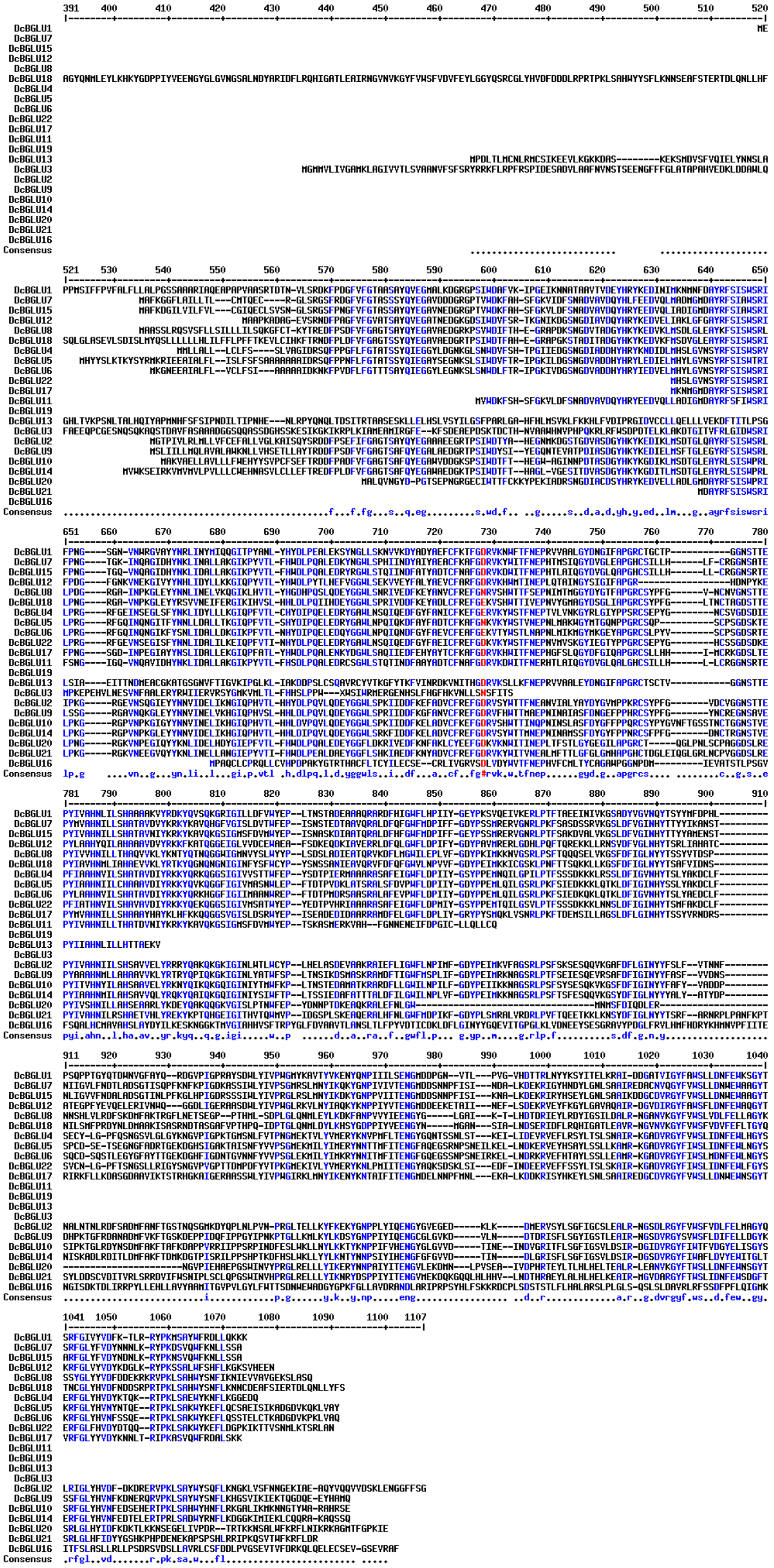

Figure S2. Amino acid sequence alignment of 22 DcBGLU enzymes in *D. catenatum*. The blue shading reflects conserved amino acid residues.

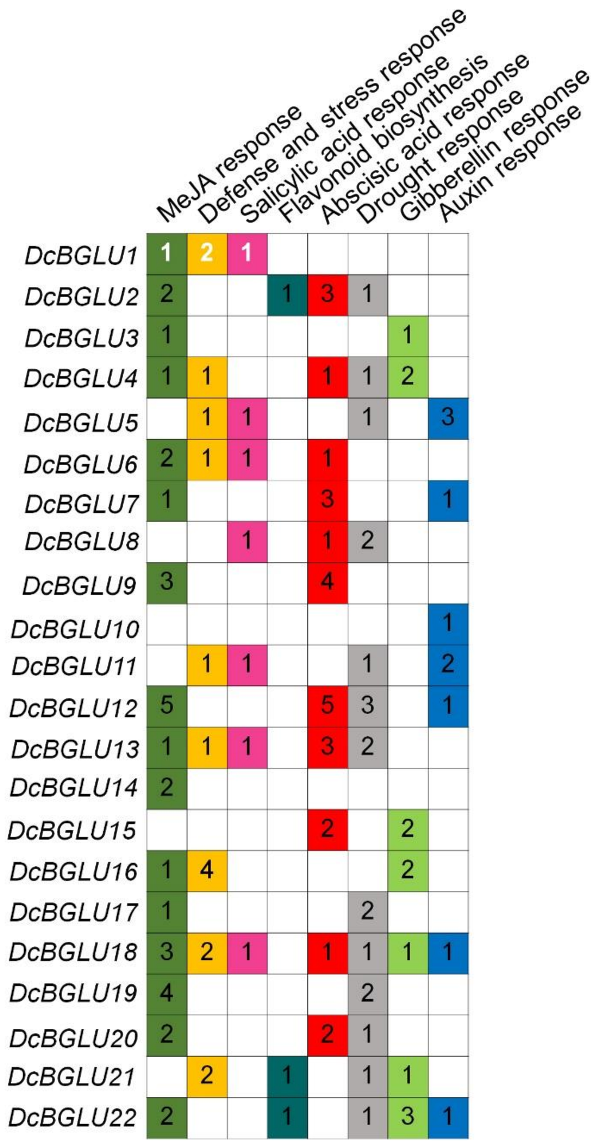

Figure S3. Analysis of the numbers and types of *cis*-acting elements in *DcBGLU* genes. Colors and numbers of the grid indicated the numbers of different *cis*-acting elements in *DcBGLU* promoters.

References

1. Wang, Z.C.; Z. M.L.; Zhang, X.J.; Zhang, Z.L.; Li, S.Z.; Li, J.; Cui, H.Q.; Li, W.J.; Liu, Y.C.; Wang, Y.; Li, L.Q.; Gu, L.L.; Wang, M.N. Phytohormone-triggered transcriptional changes revealed β-glucosidase as a key player for polysaccharide metabolism in *Dendrobium officinale*. *Prog. Biochem. Biophys.* **2022**, https://kns.cnki.net/kcms/detail/11.2161.Q.20220104.1432.004.html.
