# Supplementary figures and images for: Genome-wide identification and characterization of active ingredients related β-Glucosidases in Dendrobium catenatum
Source: BMC Genomics. 2022 Aug 23;23:612. doi: 10.1186/s12864-022-08840-x (PMC9400273; doi:10.1186/s12864-022-08840-x)

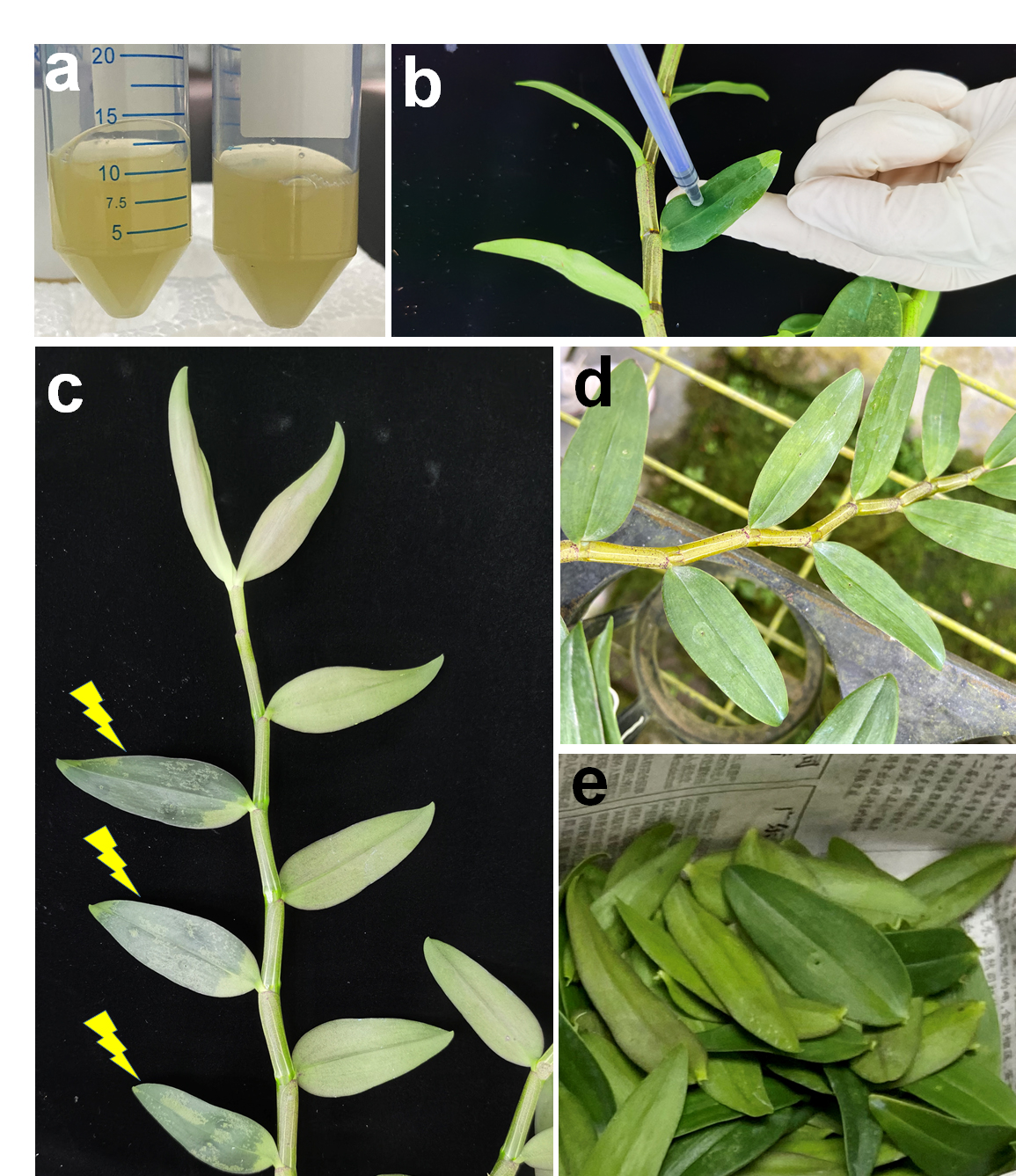

Supplement: Supplementary file 2 — Additional file 2: Fig. S1. Transient expression of DcBGLUs in leaves of D. catenatum. Fig. S2. Amino acid sequence alignment of 22 DcBGLU enzymes in D. catenatum. Fig. S3. Analysis of the numbers and types of cis-acting elements in DcBGLU genes. Table S1. Accession numbers for proteins used in the phylogenetic tree. Table S2. Primers used for qRT-PCR validation. Table S3. Primers used for gene cloning. Table S4. Abbreviations. [file 12864_2022_8840_MOESM2_ESM.zip › Fig. S1.jpg]

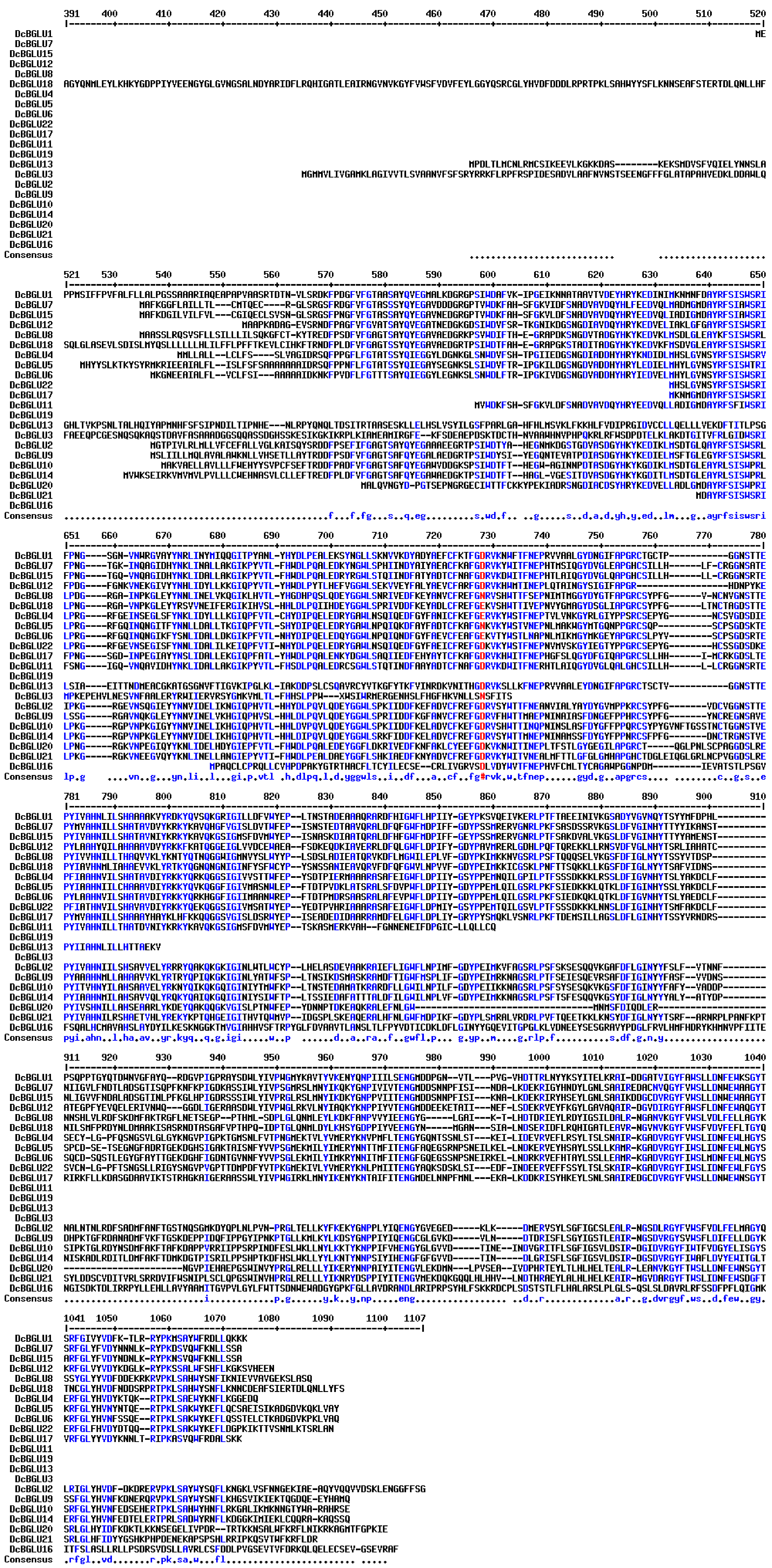

Supplement: Supplementary file 2 — Additional file 2: Fig. S1. Transient expression of DcBGLUs in leaves of D. catenatum. Fig. S2. Amino acid sequence alignment of 22 DcBGLU enzymes in D. catenatum. Fig. S3. Analysis of the numbers and types of cis-acting elements in DcBGLU genes. Table S1. Accession numbers for proteins used in the phylogenetic tree. Table S2. Primers used for qRT-PCR validation. Table S3. Primers used for gene cloning. Table S4. Abbreviations. [file 12864_2022_8840_MOESM2_ESM.zip › Fig. S2.jpg]

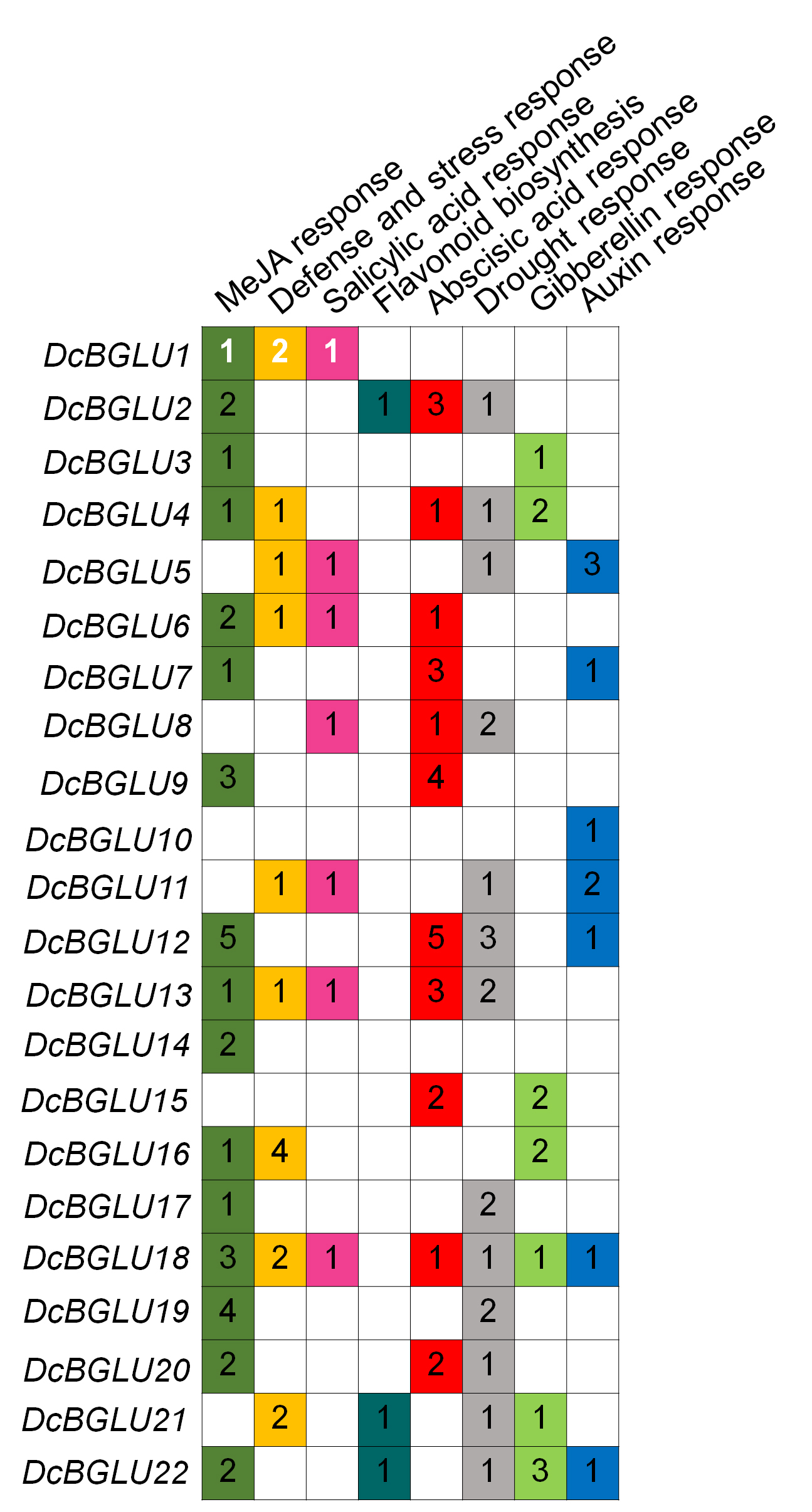

Supplement: Supplementary file 2 — Additional file 2: Fig. S1. Transient expression of DcBGLUs in leaves of D. catenatum. Fig. S2. Amino acid sequence alignment of 22 DcBGLU enzymes in D. catenatum. Fig. S3. Analysis of the numbers and types of cis-acting elements in DcBGLU genes. Table S1. Accession numbers for proteins used in the phylogenetic tree. Table S2. Primers used for qRT-PCR validation. Table S3. Primers used for gene cloning. Table S4. Abbreviations. [file 12864_2022_8840_MOESM2_ESM.zip › Fig. S3.jpg]
